# Bruxism in implant-supported rehabilitations: a narrative review of clinical complications and management strategies

**DOI:** 10.1186/s12903-025-07005-y

**Published:** 2025-10-09

**Authors:** Elnaz Shafiee, Amin Nourizadeh

**Affiliations:** 1 Department of Prosthodontics, Faculty of Dentistry, TaMS.c.,Islamic Azad University, Tabriz, Iran; 2 Department of Prosthodontics, Faculty of Dentistry, Tabriz University of Medical Sciences, Tabriz, Iran

**Keywords:** Bruxism, Dental implants, Occlusal splint, Mechanical complications, Stress distribution, Implant failure, Parafunctional habits, Biomechanics, Preventive strategies

## Abstract

**Background:**

Bruxism represents a significant risk factor for complications in implant-supported rehabilitations. Unlike natural teeth cushioned by periodontal ligament, the rigid osseointegrated connection transmits excessive bruxing forces directly to the implant-bone interface and prosthetic components.

**Objective:**

This narrative review synthesizes current knowledge regarding bruxism-related complications in implant-supported rehabilitations and evaluates available management strategies.

**Methods:**

A comprehensive narrative review was conducted using PubMed/MEDLINE, Embase, Web of Science, Scopus, Cochrane Library, and Science Direct databases covering publications from January 1990 to December 2024. Studies investigating bruxism’s relationship with dental implant outcomes, clinical complications, and management strategies were included.

**Results:**

Systematic reviews demonstrate that bruxing patients have 2.2–4.7 fold increased implant failure risk compared to non-bruxing patients. Finite element analyses reveal that occlusal splints reduce stress concentration by 33–73% depending on load magnitude. Limited evidence exists regarding the association between bruxism and peri-implant bone loss. Technical complications include prosthetic component fractures, screw loosening, and framework failures. Management strategies include occlusal splint therapy, modified prosthetic designs, strategic implant placement, and comprehensive follow-up protocols.

**Conclusions:**

While bruxism significantly increases the risk of complications in implant-supported rehabilitations, successful outcomes are achievable through comprehensive management approaches. Evidence supports the effectiveness of occlusal splints in stress reduction and splinted prosthetic designs for optimal force distribution. However, the relationship between bruxism and implant complications remains complex, requiring standardized diagnostic criteria and evidence-based treatment protocols.

## Introduction

Dental implant therapy has revolutionized the treatment of edentulous and partially edentulous patients, representing one of the most successful applications in modern dentistry. However, the success and longevity of implant-supported rehabilitations can be significantly influenced by various patient-related factors, with bruxism emerging as one of the most concerning risk factors for implant-related complications[1].

Bruxism, defined as a repetitive jaw-muscle activity characterized by clenching or grinding of teeth and/or by bracing or thrusting of the mandible, affects a substantial portion of the population and presents unique challenges in implant dentistry[2]. The prevalence of sleep bruxism in adults ranges from 8 to 16%, while awake bruxism affects approximately 22–31% of adults, making it a common condition that clinicians frequently encounter in implant treatment planning[3]. The condition can be classified into sleep bruxism and awake bruxism, with both forms potentially generating excessive occlusal forces that may compromise implant stability and prosthetic integrity.

The biomechanical implications of bruxism in implant-supported rehabilitations are particularly significant due to fundamental differences between natural teeth and osseointegrated implants. Unlike natural teeth, which are cushioned by the periodontal ligament that can absorb and redistribute occlusal forces, implants are rigidly anchored to bone through osseointegration[4]. This rigid connection means that excessive forces generated during bruxing episodes are transmitted directly to the implant-bone interface and prosthetic components, potentially leading to mechanical and biological complications.

Historically, bruxism was considered a relative or absolute contraindication for implant therapy due to concerns about increased failure rates and complications [5]. However, emerging evidence suggests a more nuanced relationship between bruxism and implant outcomes. Several systematic reviews and meta-analyses have demonstrated that while bruxism does increase the risk of implant failure and complications, it does not necessarily preclude successful implant treatment when properly managed [1, 2, 6].

The forces generated during bruxism episodes can reach 6–10 times those of normal function, placing extraordinary demands on both the implant-bone interface and prosthetic components[3]. This becomes particularly challenging in full-arch rehabilitations and complex prosthetic reconstructions, where the extensive nature amplifies the consequences of any complications.

Despite the growing body of literature on bruxism and dental implants, there remains a lack of consensus regarding optimal management strategies for patients with bruxism requiring implant-supported rehabilitations. Current approaches range from conservative measures such as occlusal splint therapy to more aggressive interventions including modified implant placement protocols and specialized prosthetic designs [4, 7, 8].

### Aim

This narrative review aims to synthesize current knowledge regarding the clinical complications associated with bruxism in implant-supported rehabilitations and to evaluate the effectiveness of various management strategies available to clinicians.

## Methods

### Search strategy

A comprehensive literature search was conducted using the PubMed/MEDLINE (National Library of Medicine), Embase (Elsevier), Web of Science (Clarivate Analytics), Scopus (Elsevier), Cochrane Library (Cochrane Collaboration) and Science Direct (Elsevier) electronic databases.

Additional searches were performed in Google Scholar for grey literature and additional relevant publications, Manual hand searching of reference lists from included studies and relevant review articles, Journal-specific searches in high-impact dental and implant journals.

### Search time frame

The literature search covered publications from January 1990 to December 2024, encompassing the modern era of dental implantology and the evolution of understanding regarding bruxism and implant complications.

#### Inclusion criteria

- Studies investigating the relationship between bruxism and dental implant outcomes.
- Research focusing on implant-supported rehabilitations (including full-arch, partial rehabilitations, and single implants).
- Clinical studies, systematic reviews, meta-analyses, and case series.
- Studies reporting complications related to bruxism in implant patients.
- Research on management strategies for bruxing patients with implants.
- Publications in peer-reviewed journals.

#### Exclusion criteria

- Single case reports.
- Animal studies.
- In vitro studies without clinical relevance.
- Studies with insufficient data on bruxism diagnosis.
- Publications not available in full text.
- Duplicate publications.

## Results

### Implant failure risk in bruxing patients

The relationship between bruxism and dental implant failure has been extensively investigated through multiple systematic reviews and meta-analyses. Zhou et al. conducted a comprehensive meta-analysis revealing that bruxing patients demonstrated significantly higher failure rates compared to non-bruxing patients, with increased odds ratios for both prostheses and patients [1]. These findings were corroborated by subsequent studies, with Häggman-Henrikson et al. reporting that implants placed in probable bruxing patients had a 2.2-fold higher risk of failure than in non-bruxing patients (OR 2.2; 95% CI 1.337, 3.583, *p* = 0.002) [6].

A more recent systematic review by Ionfrida et al. analyzed studies and found a significant pooled effect with an increased odds ratio, reinforcing the substantial impact of bruxism on implant outcomes[2]. Chrcanovic et al., in their large retrospective study of 2,670 patients with 10,096 implants, demonstrated that bruxing patients had implant failure rates of 13.0% compared to 4.6% in non-bruxing patients (*P* < 0.001), with bruxism being a statistically significant risk factor for implant failure (HR 3.396; 95% CI 1.314, 8.777; *P* = 0.012) [9].

### Biomechanical impact and stress distribution

The biomechanical impact of bruxism on implant-supported rehabilitations is complex. Finite element analysis studies have provided valuable insight into stress distribution patterns under parafunctional loading conditions. Dos Santos Marsico et al. demonstrated that the presence of occlusal splints significantly reduced stress developed in implants regardless of load condition, implant region, or connection type [10]. Their analysis showed that occlusal splints decreased stress in implants while transferring loads to bone tissues.

Silva et al. conducted a three-dimensional finite element analysis examining the biomechanical behavior of 3-unit implant-supported prostheses under parafunctional forces, finding that occlusal devices improved biomechanical behavior by reducing stress in abutment screws and stress and strain in bone tissue[11]. However, they noted that occlusal devices alone were not sufficiently effective to negate the biomechanical benefit of splinting prosthetic components.

Henrique et al. reported that oblique loading stresses significantly increased in the absence of occlusal splints, with tensile stresses on different implant systems varying by 4–19% [12]. The study demonstrated that MT implant bone tissue produced 4–19% lower stress values than HE implants, with the most significant differences observed under oblique loading conditions.

### Biological complications

#### Peri-implant bone loss

The evidence regarding the association between bruxism and peri-implant bone loss remains limited and shows only slight relationships. Bertolini et al. conducted a systematic review on traumatic occlusal forces and peri-implant bone loss, finding insufficient evidence to establish a clear causal relationship [13]. Bredberg et al. compared matched groups of bruxing and non-bruxing patients and found limited differences in marginal bone loss patterns[14]. Di Fiore et al. systematically reviewed peri-implant bone loss and overload, focusing on occlusal analysis, but found inconclusive evidence regarding the direct impact of bruxism on bone loss [15].Tabrizi et al. investigated the effect of bruxism on marginal bone levels around single tooth implants in the posterior mandible, reporting minimal associations [16].

#### Implant failure mechanisms

Biological complications associated with bruxism include accelerated marginal bone loss, peri-implantitis, and implant failure. The rigid connection between implants and bone means that excessive forces generated during bruxing episodes are transmitted directly to the implant-bone interface, potentially disturbing the adaptive capacity of peri-implant tissues.[4] This direct force transmission can lead to microdamage accumulation in the bone surrounding implants and subsequent complications.

However, researchers who evaluated the associations between bruxism and implant failures have indicated that implant failures in bruxism patients arose to a significant extent from implant fractures or fractures of implant components, representing overload of the implant body [1–5]. Manfredini et al. identified clinical factors on dental implant fractures through systematic review, demonstrating that mechanical overload plays a crucial role in implant component failure [1]. Chrcanovic et al. analyzed technical complications and risk factors for failure of combined tooth-implant-supported fixed dental prostheses, finding increased fracture rates in bruxing patients [2]. Manfredini et al. systematically reviewed bruxism as a risk factor for dental implants, highlighting the mechanical failure mechanisms [3]. Rangert et al. conducted a retrospective clinical analysis of bending overload and implant fracture, establishing the relationship between excessive forces and implant body failure [4]. Stoichkov and Kirov analyzed the causes of dental implant fracture retrospectively, confirming that overload-related fractures represent a significant failure mechanism in bruxism patients [5].

#### Technical and mechanical complications

The clinical manifestations of bruxism-related technical complications in implant-supported rehabilitations include prosthetic component fractures, screw loosening, abutment fractures, and framework failures. Chrcanovic et al. identified 98 bruxing patients among 2,670 patients and found that the bruxing group had a higher prevalence of mechanical complications compared to non-bruxing patients, with an odds ratio of implant failure of 2.71 (95% CI 1.25, 5.88) [17]. This represents the primary case-control study with a matched control group addressing technical complications.

Additional studies have reported various technical complications in bruxism patients. Studies on combined tooth-implant-supported prostheses and shorter implant-supported prostheses have also documented increased technical failure rates in patients with bruxism. Koenig et al. studied clinical behavior of zirconia restorations in patients with clinical signs of bruxism, reporting increased wear and fracture rates [18]. Levartovsky et al. reported on complete rehabilitation of bruxism patients, noting technical complications despite careful planning[19].

## Management strategies and preventive interventions

### Behavioral and psychological interventions

#### Cognitive behavioral therapy

Behavioral interventions targeting bruxism focus on awareness training and habit modification. Cognitive behavioral therapy (CBT) approaches include biofeedback, relaxation techniques, and sleep hygiene education [20]. While evidence specifically for implant patients is limited, general bruxism management studies suggest moderate effectiveness in reducing grinding episodes and associated symptoms[21].

#### Stress management

Since psychological stress is a significant contributing factor to bruxism, stress management techniques including mindfulness meditation, progressive muscle relaxation, and counseling may provide adjunctive benefits [22]. The integration of behavioral interventions with dental management remains underexplored in implant populations [23].

#### Sleep disorder management

Sleep-related bruxism may be associated with sleep-disordered breathing and other sleep disorders. Comprehensive sleep evaluation and management of underlying sleep disorders may reduce bruxism intensity, though specific evidence in implant patients is lacking [24].

### Pharmacological approaches

#### Muscle relaxants

Pharmacological interventions include muscle relaxants such as benzodiazepines, though their long-term use is limited by side effects and dependency concerns [25]. Short-term use may be beneficial during acute phases or initial implant healing periods [26].

#### Botulinum toxin

Botulinum toxin injections into masticatory muscles have shown promise in reducing bruxism intensity and associated muscle pain [27]. However, potential effects on chewing function and the need for repeated injections limit widespread application. Specific studies in implant patients are minimal [28].

#### Other pharmacological agents

Various medications including anticonvulsants, antidepressants, and dopaminergic agents have been investigated for bruxism management with varying degrees of success [29]. The evidence base remains limited, and potential interactions with implant healing require consideration [30].

### Dental interventions

#### Occlusal splint therapy

Occlusal splint therapy represents the primary non-invasive intervention for managing bruxism in implant patients. Clinical studies supporting the use of occlusal splints in bruxism patients with implants include the work by Sutthiboonyapan and Wang, who emphasized that occlusal splints appear effective in reducing symptoms related to temporomandibular disorder and bruxism, though they noted that high-quality evidence is lacking to support their use [31]. They proposed the use of occlusal splints as a non-invasive and reversible therapy for patients with bruxism.

Teixeira et al. used photoelastic analysis to evaluate stress distribution in implant-supported prostheses with and without occlusal splints under different loading conditions (300, 600, and 900 N) [32]. Their results showed that occlusal splint use reduced tension by 33.22%, 66.66%, and 73.33% respectively, with the protective effect becoming more evident at higher load magnitudes.

The biomechanical benefits of occlusal splints have been consistently demonstrated across multiple finite element analysis studies. Henrique et al. found that flat occlusal splints reduced stress concentration in implant prosthesis treatments, with significant increases in oblique loading stresses observed in their absence [12]. Similarly, Batista et al. demonstrated that acrylic occlusal devices were effective in reducing stress in external hexagon implants under parafunctional loading conditions [33].

#### Prosthetic design considerations

The design of implant-supported prostheses must account for the increased forces generated during bruxing episodes. Splinted prosthetic designs have consistently shown superior biomechanical performance compared to non-splinted alternatives, as supported by systematic reviews and meta-analyses (Table-1). De Souza Batista et al. conducted a systematic review and meta-analysis on splinted versus nonsplinted adjacent implants, concluding that splinting provides biomechanical advantages [34]. Ravida et al. developed a decision tree for selection between splinted and nonsplinted implants based on biological and technical considerations [35]. Shah et al. performed a systematic review comparing marginal bone loss, survival rates, and prosthetic complications between splinted and nonsplinted implant restorations [36]. According to their results, splinted implant restorations lost less bone than non-splinted implant restorations and lower implant failure was associated with splinted restorations. Restorations with and without splinting had the same level of prosthetic problems.

**Table 1:** Summary of management strategies and evidence

Management Strategy	Specific Interventions	Evidence/Effectiveness	References
Occlusal Splint Therapy	Flat occlusal splints (2 mm thickness)	Stress reduction: 33–73% depending on load	16, 13, 11
	Acrylic occlusal devices (AOD)	Significant stress reduction in external hexagon implants	17, 12
	Rigid occlusal stabilization appliances	Highly indicated for bruxism control	15, 21
Prosthetic Design Modifications	Splinted vs. non-splinted prostheses	Superior biomechanical performance with splinting	12, 17
	Material selection	Metal restorations preferred over porcelain	29
	Framework design	Rigid framework connections	Study findings
Implant Placement Strategies	Adequate implant length and diameter	Reliable treatment with reduced failure risk	15
	Increased implant numbers	Better force distribution	7
	Strategic implant positioning	Optimal force distribution patterns	6
Occlusal Scheme Modifications	Mutually protected occlusion	Recommended for implant prostheses	7, 8
	Reduced cantilevers	Minimize overloading factors	6, 7
	Narrowed occlusal table	Reduced lateral forces	7
	Increased contact points	Better force distribution	7
Connection System Selection	Internal vs. External hexagon	Internal hexagon shows advantages	13
Diagnostic Protocols	Standardized bruxism assessment	“Possible,” “probable,” “definite” grading	9, 10
	TMD symptom evaluation	Comprehensive assessment approach	18
Progressive Loading Protocols	Gradual force application	Recommended for poor bone quality	7
Follow-up and Monitoring	Regular maintenance protocols	Essential for long-term success	4, 15
	Parafunction monitoring	Continuous assessment	Multiple studies
Reverse Planning Approach	Occlusal splint before restoration	Comprehensive treatment strategy	20
Multidisciplinary Management	Integrated treatment approach	Comprehensive patient care	19, 21

Silva et al. demonstrated that splinted crowns in the posterior maxillary region with occlusal devices represented the most effective method of reducing stress in abutment screws and stress and strain in bone tissue under parafunctional conditions [11]. Material selection also plays a crucial role in managing bruxism-related complications, with metal restorations often preferred over porcelain for patients with presumed bruxism based on tooth wear patterns.

#### Strategic implant placement

Strategic implant placement and increased implant numbers can help distribute occlusal forces more effectively in bruxism patients. Sarmento et al. emphasized that rehabilitation of bruxing patients using implant-supported prostheses with adequate implant length and diameter, as well as proper positioning, appears to be a reliable treatment approach with reduced failure risks [37] .

#### Occlusal considerations and schemes

The optimal occlusal scheme for implant-supported rehabilitations in bruxism patients remains a subject of ongoing research. Goldstein et al. conducted a systematic review of occlusal schemes for implant restorations and found limited scientific evidence regarding optimal approaches [38]. Kim et al. provided clinical guidelines for implant occlusion with biomechanical rationale, emphasizing that dental implants may be more prone to occlusal overloading due to the absence of periodontal ligament [7]. Sheridan et al. recommended a mutually protected occlusion with anterior guidance and evenly distributed contacts with wide freedom in centric relation for implant-supported restorations^⁷^.

#### Diagnostic considerations

Accurate diagnosis of bruxism remains challenging due to the episodic nature of the condition and varying diagnostic criteria. Häggman-Henrikson et al. emphasized the importance of using studies that provided information on self-report and clinical examination needed for the diagnosis of at least “probable” bruxism [6]. The diagnostic grading system of “possible,” “probable,” and “definite” sleep or awake bruxism, based on international consensus criteria, provides a standardized approach for clinical assessment.

It is important to note that bruxism is a risk factor for temporomandibular disorders (TMD) but is not synonymous with TMD. Chatzopoulos and Wolff investigated the association between symptoms of temporomandibular disorder and self-reported bruxism with implant failure risk, finding no significant association between bruxism symptoms and implant failure, highlighting the complexity of the relationship [39].

#### Treatment planning considerations

Treatment planning for implant-supported rehabilitations in bruxism patients requires a multidisciplinary approach. Manfredini and Poggio conducted a systematic review of prosthodontic planning in patients with temporomandibular disorders and/or bruxism, finding an absence of randomized controlled trials on various topics concerning the relationship between TMD, bruxism, and prosthodontics [40] .

The concept of reverse planning, as described by Dias et al., involves creating an occlusal splint before restorative treatment, ensuring the reestablishment of shape, function, and dental aesthetics while controlling the cause of the wear process [41].This approach represents a comprehensive strategy that addresses both the protective and restorative aspects of treatment.

#### Long-term management and follow-up

Long-term management of bruxism patients with implant-supported rehabilitations requires ongoing monitoring and maintenance protocols. The importance of regular follow-up and monitoring cannot be overstated. Komiyama et al. noted that although it is clear that implant-supported rehabilitation of patients with bruxism requires adequate planning and follow-up, well-designed randomized controlled trials are needed to provide reliable evidence on the long-term success of this treatment modality [4].

## Discussion

The evidence clearly demonstrates that bruxism represents a significant risk factor for complications in implant-supported rehabilitations, with 2.2–4.7 fold increased risk of implant failure compared to non-bruxing patients [1, 2, 6].However, this increased risk does not preclude successful treatment when appropriate management strategies are implemented.

The relationship between bruxism and peri-implant bone loss appears to be more complex than initially assumed, with only limited evidence showing slight associations [13–16]. This finding challenges the traditional understanding and suggests that other factors may play more significant roles in biological complications around implants in bruxism patients.

Technical and mechanical complications represent the primary concern in bruxism patients with implant-supported rehabilitations. The evidence base for technical complications, while limited to primarily one high-quality case-control study, suggests significantly higher rates of prosthetic component failures, screw loosening, and framework fractures in bruxism patients [17].

Management strategies show varying levels of evidence support. Occlusal splint therapy has proven effective in reducing stress concentration by 33–73% depending on load magnitude, with consistent results across multiple biomechanical studies [10–12, 32, 33].However, clinical studies supporting splint therapy remain limited. Splinted prosthetic designs provide superior biomechanical performance under parafunctional loading conditions, supported by high-quality systematic reviews and meta-analyses [34, 35].

The diagnostic challenge of bruxism remains significant, with the need for standardized criteria and reliable assessment methods. The current grading system of “possible,” “probable,” and “definite” bruxism provides a framework, but clinical implementation remains inconsistent [6].

Future research directions should focus on well-designed randomized controlled trials evaluating long-term outcomes of various management strategies, development of standardized diagnostic criteria, and investigation of the complex relationship between bruxism, implant complications, and patient-specific factors.

Several significant limitations exist in the current evidence base:

1. *Diagnostic Standardization*: Bruxism diagnosis remains inconsistent across studies, with varying criteria and assessment methods limiting comparability of results.
2. *Study Design Limitations*: Most evidence comes from retrospective studies and finite element analyses, with limited prospective clinical trials specifically designed for implant patients with bruxism.
3. *Behavioral Management Evidence*: While behavioral interventions show promise in general bruxism management, specific evidence for their effectiveness in implant patients is minimal.
4. *Long-term Outcomes*: Limited long-term follow-up studies hamper understanding of the durability of various management strategies.
5. *Patient-Specific Factors*: Individual variations in bruxism intensity, implant site factors, and patient compliance are poorly characterized in existing literature.

## Conclusions

While bruxism significantly increases the risk of complications in implant-supported rehabilitations, successful outcomes are achievable through comprehensive management approaches [42]. Evidence supports the effectiveness of occlusal splints in stress reduction and splinted prosthetic designs for optimal force distribution. However, the relationship between bruxism and implant complications remains complex, with limited evidence for associations with peri-implant bone loss and primarily technical complications being well-documented.

Successful management requires accurate diagnosis, strategic treatment planning, appropriate prosthetic design, occlusal splint therapy, and long-term monitoring. Continued research is needed to establish evidence-based protocols and optimize treatment outcomes for this challenging patient population.

The emerging recognition of behavioral and pharmacological interventions highlights the need for interdisciplinary approaches. While dental interventions address the mechanical aspects of force management, addressing the underlying bruxism behavior may provide more comprehensive long-term benefits.

Clinical Recommendations:

1. Systematic bruxism assessment using standardized diagnostic criteria.
2. Implementation of occlusal splint therapy as primary protective intervention.
3. Preference for splinted prosthetic designs in multi-unit rehabilitations.
4. Strategic implant placement with adequate number and positioning.
5. Comprehensive long-term monitoring and maintenance protocols.
6. Multidisciplinary treatment planning approach.

## Data Availability

The datasets used and/or analyzed during the current study are available from the corresponding author on reasonable request.
